# Epigenetic erosion of H4K20me1 induced by inflammation drives aged stem cell ferroptosis

**DOI:** 10.21203/rs.3.rs-3937628/v1

**Published:** 2024-02-14

**Authors:** Romeo Blanc, Nidhi Shah, Noah Salama, Fanju Meng, Alireza Mousaei, Joe Chakkalakal, John Onukwufor, Patrick Murphy, Laura Calvi, Robert Dirksen

**Affiliations:** university of rochester; university of rochester; university of rochester; university of rochester; university of rochester; university of rochester; university of rochester; university of rochester; university of rochester; University of Rochester

## Abstract

Aging is associated with a decline in stem cell functionality and number across the organism. In this study, we aimed to further unravel Muscle Stem Cells (MuSCs) aging by assessing how systemic factors influence MuSC fate decisions through long-term epigenetic landscape remodelling. As aging is intricately linked to a pro-inflammatory shift, we studied the epigenetic effects of inflammatory signals in MuSCs and measured decreased H4K20me1 levels. This loss disrupts MuSC quiescence, largely through epigenetic silencing of Notch target genes. In the setting of inflammatory signals or aging, the lack of Kmt5a and the subsequent absence of *de novo* H4K20me1 culminate in cell death by ferroptosis. Aged MuSCs manifest abnormal iron metabolism and reduced Gpx4 levels, resulting in the accumulation of intracellular iron, increased reactive oxygen species, genomic instability, and lipid peroxidation. We showed that ferroptosis is the predominant mode of cell death in aged MuSCs, with remarkably high levels of lipid peroxidation; a phenomenon we also observed in aged hematopoietic stem cells. Implementing preventative strategies to inhibit systemic inflammation prevented aged MuSC ferroptosis, preserving their numbers and regenerative capabilities. This intervention significantly enhanced aged muscle regeneration and strength recovery and extended both lifespan and healthspan in mice. This study delineates a previously underappreciated fate trajectory for stem cell aging, and offers meaningful insights into the treatment of age-related disorders.

## Introduction

Aging is a complex biological process characterized by intricate cellular changes, central to which adult stem cells are crucial for maintaining tissue homeostasis. With age, the functionality of these stem cells markedly declines, leading to a plethora of physiological alterations. Aged skeletal muscles are plagued by decreased muscle mass and function and impaired regenerative capacity, which correlates with the loss of muscle stem cells (MuSCs) and increased inflammation^1–3^. Although the relationship between these events has been extensively studied, the molecular mechanisms underlying them remain unclear.

A core concept of aging is the loss of epigenetic information, with erosion of histone modifications serving as one of its central axes^4,5^. Histone modifications have been scarcely studied in MuSCs but have been identified as critical determinants of MuSC quiescence and activation^6,7^. Although previous studies have highlighted the role of H4K20 in adult stem cell function, the exact contribution of H4K20 during the aging process remains unknown^8,9^. In addition, identifying the mechanisms underlying epigenetic erosion remains a challenge in the study of stem cell aging. Growing evidence indicates the contribution of several extrinsic factors such as inflammation^10^.

With age, MuSCs lose quiescence and transition toward irreversible fates, such as death or senescence, leading to defective muscle repair^1,11–13^. In the wake of the first rejuvenation experiments using heteroparabiosis^14,15^, few studies have investigated how systemic molecular and cellular changes might affect stem cell rejuvenation, focusing instead on the stem cell niche, thus leaving a knowledge gap. In this study, we aimed to further unravel the mysteries of MuSC aging by assessing how systemic factors influence MuSC fate decisions through long-term epigenetic landscape remodelling. By employing a comprehensive approach combining epigenomics, single-cell measurements, and functional testing of MuSCs from young and old mice, we investigated the molecular mechanisms underlying age-related defects in the fate, functionality, and survival of MuSCs. We present evidence that age-associated inflammation induces profound epigenetic remodeling in MuSCs, culminating in their premature exit from quiescence and susceptibility to an iron-dependent form of cell death termed ferroptosis. Our data underscores that acute systemic inflammation distinctly targets histone H4 lysine 20 methylation, subtly reconfiguring the MuSC transcriptome to a state that anticipates subsequent injury responses. During aging, chronic low-grade systemic inflammation results in compromised activity of lysine methyltransferase 5a (Kmt5a), thereby disrupting the crucial transcriptional programs necessary for preserving MuSC quiescence and survival.

## Results

### Age-associated systemic inflammation and MuSCs epigenetic remodelling

**Figure 1 -:**
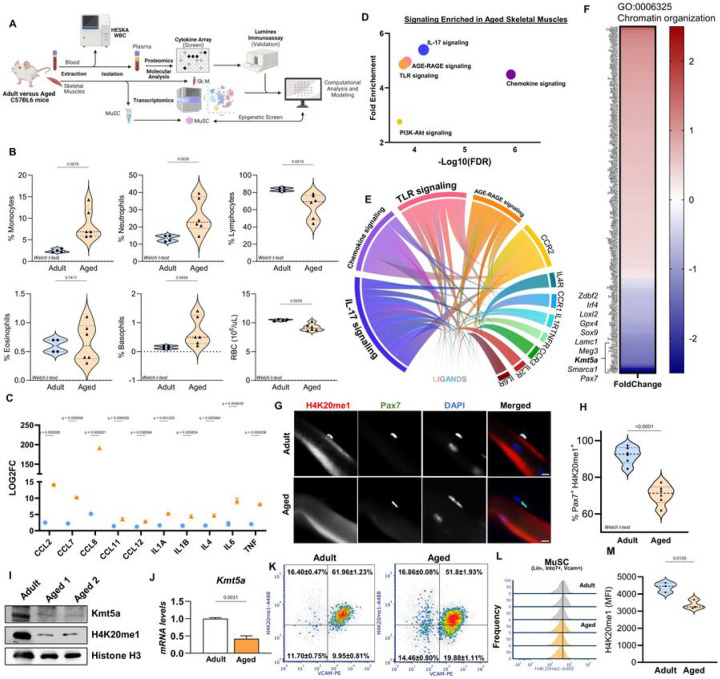
Age-associated systemic inflammation correlates with chemokine signalling activity in skeletal muscles and chromatin remodelling in MuSCs. **A-F)** Schematic for multiomics data integration and subsequent computational modelling of inflammatory signaling in skeletal muscles by systemic ligands (A). Blood was collected to assess cellular content contributing to the inflammatory phenotype in aged mice (B) followed by plasma collection (n = 6 mice per conditions). Proteomics data from plasma were first used to identify cytokines enriched with aging using a large array profiler followed by immunoassay validation (C) (n = 3 mice per conditions). Transcriptomics from whole skeletal muscles were used to identify potential activated signaling based on expression changes of the genes within this signaling (D). Both dataset were integrated through a scoring system to predict ligand-receptor interaction that could account for the activated signaling in skeletal muscles (E).The analysis pointed to IL-17 and chemokine signaling to be the most activated signaling, with CCR2 identified as the top upstream regulator of these signaling through ligands-receptor interaction. **F)** Heatmap for genes associated with Chromatin organization (G0:0006325). This term was the most enriched epigenetic-associated term when comparing transcriptome changes between adult and aged MuSCs. **G, H)** Representative pictures (G) and quantification (H) of H4K20mel status in MuSCs on EDL single fibers isolated from adult and aged mice (n = 6 mice per age group; with at least 30 Pax7+ cell counted per mouse). I. J) Immunoblot (I) and qPCR (J) assessing KmtSa mRNA and protein levels from freshly sorted muscle stem cells (n 3 mice per condition). Low Kmt5a correlated with decreased H4K20mel. For western blots, two aged mice were pulled together for one lane. Quantification is normalized to total 113 and relative to young values. **K-M)** Flow cytometry analysis for MACS prc-sortcd cells stained for VCAM and Zombie dye prior fixation, then fixed and pcrmeabilized for H4K20mcl staining. Graph display percentage of cells positive for VCAM and H4K20mel for n = 3 micc per age). 21) plot of H4K20mcl in MuSC for each rcplicatc (L) and mean fluorescence intensity analysis (M) (n = 3 mice per age). For all violin plots, data represent biological rcplicates. Statistical significance was determined using Welch’s t-tcst and exact adjusted p-valucs are reported in the figure.

To better understand the intricate relationship between systemic aging and MuSCs, we conducted a comprehensive analysis of the molecular and cellular characteristics of the murine aging circulatory system and compared them with both aged MuSC intrinsic changes and aged MuSC niche extrinsic changes (Fig. 1A). First, we used whole blood counts followed by plasma collection to analyze the molecular and cellular blood profiles of young and aged mice. To ensure an unbiased approach, we first screened over 100 cytokines in young and aged mice using a proteome profiler array, followed by secondary validation of the identified candidates using quantitative multiplexing immunoassays. As expected, the plasma from aged mice displayed elevated levels of circulatory cytokines (TNFa, IL1a, IL1b IL-4, IL-6, CCL2, CCL7, CCL11, and CCL12) and myeloid cells, and a decline in lymphoid cells (Fig. 1B, 1C, and Table S1). Together, these data support the widely recognized age-associated chronic low-grade inflammation, often referred to as “*inflammaging*,” and myeloid bias resulting from the aging hematopoietic lineage. Secondly, when we interrogated the aged skeletal muscle transcriptome using our previously published dataset^16^, we found that the top three enriched activated pathway (Z-score > 2) were “Chemokine signaling”, “Cytokine-Cytokine Interaction” and “Complement & Coagulation Cascades” (Table S1). This led us to wonder whether increased circulatory cytokine levels could affect aged skeletal muscle. Using a suite of computational analyses, we integrated our proteomics data with transcriptomic results to generate a ligand-receptor predictive model and the subsequent signaling activity^17^. We identified five signaling predicted to be activated by our ligand-receptor model, including “Chemokine Signaling”, “IL-7 signaling” and “TLR-signaling” (Fig. 1D and E and Table S1). Interaction analysis predicted CCR2 to have the highest activation score, significantly contributing to all but one of the signaling pathways (Fig. 1E).

We previously established that CCR2 signaling activation is a hallmark of skeletal muscle aging^1^. The levels of circulatory CCR2-ligands have also been thoroughly characterized as a measure of biological age, frailty, and age-associated systemic inflammation in both humans and mice^11,18–20^. Building on this foundation, our first objective was to elucidate the connection between systemic inflammation and the intrinsic changes observed in skeletal muscles and MuSCs with advancing age. We first simulated a directed CCR2-mediated inflammatory response by systemically injecting recombinant CCL2, CCL7, and CCL8 into young animals as previously described^1^. Following injection, we recorded a transient inflammatory response, as evidenced by the increase in both the number of monocytes and the levels of pro-inflammatory cytokines in the blood of the treated mice (Figure S1E-J). We also assessed how this acute inflammatory response affects MuSCs, using CCR2-null mice as a negative control^1^. Although we observed no change in the raw number of MuSCs (Pax7+), stem cells from mice injected with chemokines displayed enhanced activation (Ki67+), cell cycling (EdU+), and accelerated myogenesis (MyoD/MyoG+) (Figure S1A-D). These have been previously described as features of the G_alert_ state^21,22^. Interestingly, these enhanced characteristics remained long after chemokine levels returned to baseline (Figure S1K-M), whereby we observed accelerated regeneration (Figure S1N-P), in line with previous reports regarding G_alert_. MuSCs that have experienced prior injury have a long-term enhanced regenerative capacity^23^, yet the mechanisms underlying this phenomenon are not well understood. These long-term functional changes prompted us to believe that MuSCs can adapt to acute inflammatory signals, which are indicative of epigenetic reprogramming^24,25^.

We hypothesized that age-associated systemic inflammation might affect the epigenome and transcriptome of MuSCs. Following our results, we performed an in-depth examination of the aged MuSC transcriptome and found that one of the most significantly enriched pathways was “Chromatin organization” (Fig. 1F), in with our initial hypothesis and previous reports^7,26^. Of particular interest was the lysine methyltransferase Kmt5a, because of its crucial role in maintaining H4K20 methylation. Kmt5a-mediated catalysis of H4K20 monomethylation is required for subsequent di- and trimethylation, which is necessary for establishing constitutive heterochromatin. The dynamic regulation of both Kmt5a and H4K20me1 is also indispensable for proper cell cycle progression, which is intriguing given that MuSCs predominantly exist in a state of quiescence and aged MuSCs often die upon activation. We confirmed our RNA-seq results to show that both the Kmt5a gene and protein decreased in aged MuSCs (Fig. 1I and J). Loss of Kmt5a was functionally reflected by decreased methylation of its main substrate, H4K20me1 (Figure G-M). Single-cell analysis revealed a shift in H4K20me1 intensity in the aged MuSC population.

Next, we assessed whether CCR2 activity is linked to *Kmt5a* repression. In response to the systemic delivery of CCR2-ligands, *Kmt5a*, but no other epigenetic genes, was significantly repressed in MuSCs (Figure S1Q and R). It was also accompanied by long-term transcriptional changes in both myogenic and cell cycle genes (Figure S1S and S1T). Notably, we observed the upregulation of activation genes (*Myf5* and *Dek*) and downregulation of quiescence genes (*Hes1* and *Hey1*). We found that MuSCs displayed long-term erosion of H4K20me1 and a sustained increase in MyoD + cells lasting for at least six-weeks post-injection (Figure S1U-X). Single-cell analysis also pointed that CCR2-ligands treatment promoting a shift in H4K20me1 intensity, with the population of MuSCs derived from treated animals displaying lower H4K20me1 levels (Figure S1W and S1X). These results suggest that CCR2-mediated inflammation may trigger long-term epigenetic remodelling in MuSCs, which is potentially mediated by Kmt5a and H4K20 methylation. Thus, we further investigated the role of Kmt5a in MuSCs to better understand its effect on muscle aging.

### Kmt5a is required for quiescence maintenance and MuSC survival after activation.

To assess the role of Kmt5a in quiescent MuSCs, we generated inducible *Kmt5a* MuSC-specific knockout mice (Pax7^CreERT2/+^; Kmt5a^fl/fl^ [Kmt5a^KO^]) (Figure S2A). Immediately following the deletion of *Kmt5a*, we did not observe a change in MuSC number *in vivo*, despite the vast majority of Kmt5a^KO^ MuSCs lacking Kmt5a (> 95% efficiency; orange arrows) (Figure S2B-D). Next, we cultured both wildtype (Pax7^+/+^; Kmt5a^fl/fl^) and Kmt5a^KO^ mice to assess myogenic potential, cell proliferation, and survival. Freshly isolated MuSCs (Pax7+) were stained with myogenic markers to assess myogenesis progression toward commitment (MyoD) and terminal differentiation (MyoG) over time^1^. Ki67 and EdU were used to assess cell cycle re-entry (activation) and active cell proliferation, respectively. When we challenged the cells outside their niche, cultured *Kmt5a*-null cells displayed enhanced activation (Pax7 + Ki67+), which directly correlated with the loss of H4K20me1 as early as 24hours after plating (Figure S2E-I). However, once MuSCs started actively dividing (EdU+) after 72h of culture, we observed a near-complete loss of H4K20me1, which is in line with the cell cycle regulation of H4K20me1. This decrease was accompanied by impaired myogenic terminal differentiation and cell expansion (Figure S2E-K). Further analyses revealed that Kmt5a^KO^ MuSCs displayed aberrant morphological phenotypes, such as blebbing, pycnotic, and multiple nuclei, and a molecular signature suggestive of genomic instability (Figure S2L, M; red arrows). Molecular analysis of 72h cultured MuSCs supported this premise, as mutant cells displayed elevated y-H2AX and serine 15 phosphorylation of P53.

**Figure 2 -:**
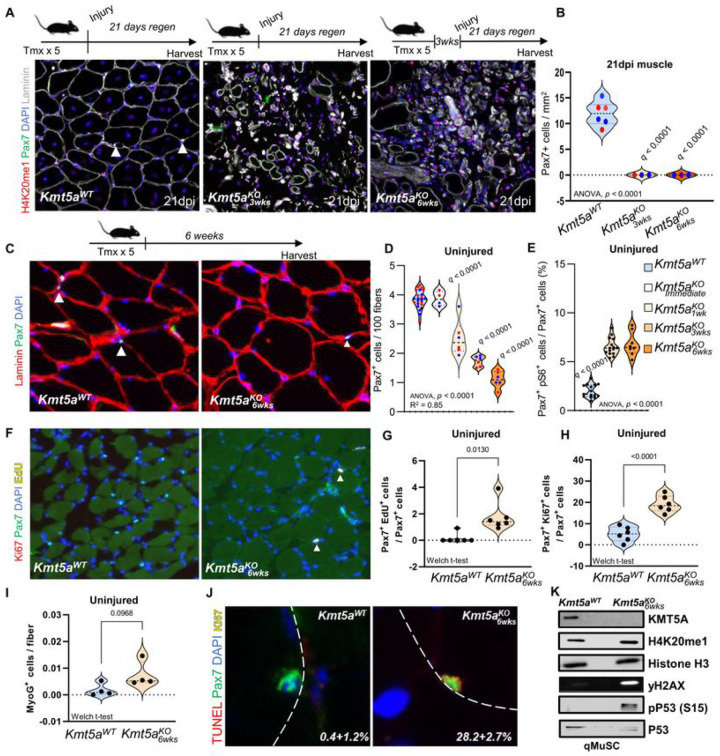
KmtSa is required for quiescent muscle stem cell homeostasis. **A, B)** Schematic representation of the regeneration experiments and muscle stem cell self-renewal. Mice were subjected to injury either immediately after tamoxifen injections or three weeks later. Injured muscles were harvested 21 days post-injury, and muscle stem cells were counted by staining for Pax7^+^. The relative number of muscle stem cells per area of regeneration is shown in (B). **C, D)** Schematic representation of long-term *Kmt5a* deletion and quantification of muscic stem cell pool maintenance at homeostasis. Muscles were harvested at different times post-knockout induction, and the number of Pax7^+^ cells was countcd. The stochastic decline of muscle stem cells in the mutant mice is shown in (D). E, Percentage of G_alert_ muscle stem quantified by pS6 immunostaining three and six weeks post-knockout induction. **F-H)** Relative number of muscle stem cells actively cycling (g; EdU^+^) or out of quiescence (h; Ki67^+^) in uninjured muscles. **I)** Quantification of precocious terminally committed muscle stem cell progenitors (Pax7^+^ MyoG^+^) six weeks post-deletion of *Kmt5a* in MuSCs. **J, K)** Molecular profiling of DNA damage and DNA repair signatures in muscle stem cells six weeks post-knockout induction. TUNEL was used to detect DNA strand breaks (J) and immunoblotting was used to measure the p53 signaling response (p-P53) to DNA damage (yH2AX). For all violin plots, data are representing biological replicates. For all violin plots, data represent biological replicates. Statistical significance was determined using Welch’s t-test (G, H, 1) or one-way ANOVA (B. D, E), and exact p-values and adjusted p-values (q) arc reported in the figure.

When challenged by acute sterile injury, the contribution of Kmt5a^KO^ MuSCs to regeneration was disrupted, resulting in the apparent absence of muscle regeneration, as we did not observe centrally nucleated fibers (CNF) among the injured mutants (Fig. 2A). We also noted a macroscopic decrease in injured muscle size 21-day post-injury (dpi) (Figure S3A, B). To better index regeneration, we used embryonic myosin heavy chain (eMHC), a marker of immature (regenerating) skeletal muscle fibers, throughout the regeneration process (Figure S4A). As expected, wild-type regenerating fibers were nearly all positive for eMHC at early regeneration time points (4- and 7dpi), followed by the generation of large centrally nucleated fibers (CNF), a hallmark of regeneration. In contrast, we did not observe any regenerating fibers in Kmt5a^KO^ mice past 7dpi (Figure S4B). By 60dpi, most of the injured area appeared to be reduced to remnants of the ECM and mononucleated cells. Additionally, virtually no surviving MuSCs were observed after regeneration (Fig. 2B and S4C). When we investigated the fate of these MuSCs and their derived progenitors, we found a sharp decline in the number of myoblasts (MyoD+) in the mutant at 4dpi (Figure S4D–G). The number of terminally committed progenitors (MyoG+) briefly increased at 4dpi before virtually disappearing at 7dpi (Figure S4E-F). Next, we aimed to further understand the fate of Kmt5a^KO^ MuSCs in response to direct and indirect environmental pressures. Since MuSCs and derived progenitors were lost between 4- and 7dpi, we assessed proliferative and myogenic capacity at 4dpi in both injured and uninjured contralateral (CL) limbs, where MuSCs are expected to enter the G_alert_ state (Figure S4A)^21^. While MuSC numbers in Kmt5a^KO^ mice rapidly declined in both injured and CL muscles (Figure S4C, S4I, and S4K), MuSCs derived from the injured limb displayed primed features reminiscent of G_alert_, including swifter entry into the cell cycle (Figure S4J and S4L), accelerated myogenic progression (Figure S4E-G), and high p-S6 levels compared to wild-type MuSCs (Figure S4M-O)^22^.

Previous reports have shown that H4K20me1 levels are regulated in a cell cycle-dependent manner in some somatic cells^27^. Based on our observations, H4K20me1 levels in quiescent MuSCs were relatively stable and fell mostly after the first cell division, which occurs approximately 60h post-activation^21^ (Figure S2E and S2O, P). We hypothesized that long-term loss of Kmt5a would eventually disrupt H4K20me1 maintenance by preventing *de novo* deposition of H4K20me1 and potentially altering the capacity of MuSCs to remain quiescent. To test this theory, we deleted *Kmt5a* and waited for 1, 3, or 6 weeks before assessing MuSC number and function *in vivo* (Fig. 2C–O). Over time, the MuSC number progressively declined in a stochastic manner (Fig. 2D). While MuSCs were nearly undetectable after six weeks, the few remaining MuSCs still displayed detectable levels of H4K20me1 (Figure S2O, P). Taken together, these results support our theory that the loss of H4K20me1 over time following *Ktmt5a* deletion does not occur immediately and simultaneously in every cell. Notably, from three weeks and onward, Kmt5a^KO^ MuSCs displayed features of quiescence exit, depicted by elevated levels of pS6 (Fig. 2E) and a higher number of Ki67-positive cells (Fig. 2F,G), but a low number of actively cycling cells (EdU+) (Fig. 2F, H). Next, we investigated the fate of the Kmt5a^KO^ MuSCs *in vivo*. A previous report has demonstrated that tempering with H4K20me2 leads to spontaneous and precocious differentiation of MuSCs during homeostasis^9^. However, we did not find MyoG + cells in our mutants in homeostasis (Fig. 1I). Since we observed a decline in survival and genomic instability in cultured Kmt5a^KO^ MuSCs, which correlated with the loss of H4K20me1 (Figure S2E-N), we tested the possibility that Kmt5a^KO^ MuSCs exited quiescence and entered cell death. We found a significant portion of mutant cells showing signs of cell death (~ 28% TUNEL+) (Fig. 1J), together with an increased DNA damage response depicted by increased yH2AX and p53 signaling (Fig. 1J, K).

Taken together, these results suggest that H4K20me1 regulation is an important factor in fine-tuning MuSC quiescence and, consequently, ensuring MuSC survival in response to extrinsic cues.

### Kmt5a maintains MuSC quiescence by epigenetically regulating Notch signaling through promoter-proximal pausing.

**Figure 3 -:**
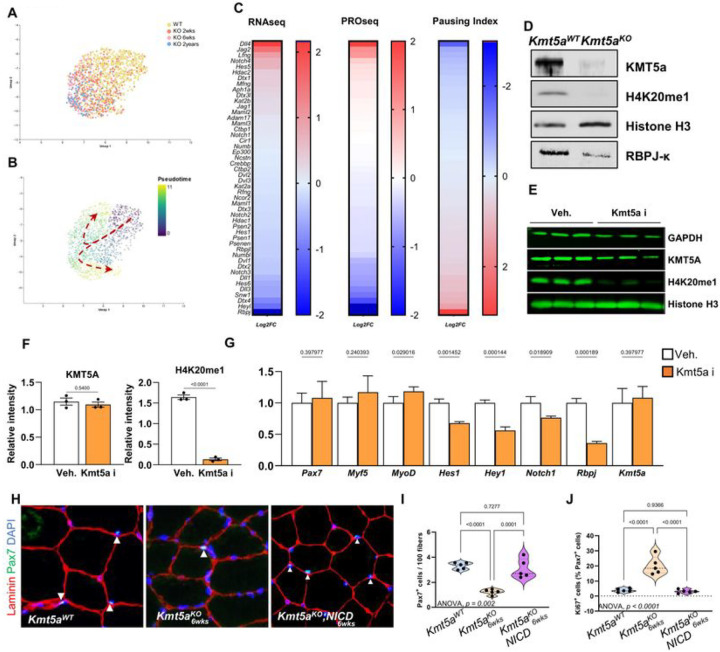
Single-cell analysis of Kmt5a^KO^ aging reveal Kmt5a as Notch master regulator. **A, B**, Embedding plot of single-cell RNA sequencing samples (A) and subsequent pseudotime analysis (B) for muscle stem cells identifying progressive shifts in the knockout populations with age (two weeks, 6weeks. and 2ycars post tamoxifen injection). **C**, Transcriptional activity of Notch signaling in response to *Kmt5a* deletion after six weeks. RNA sequencing measured total mRNA, while PRO sequencing measured nascent mRNA and precisely mapped transcriptionally-engaged RNA Pol II. Pausing index represents the ratio of promoter-proximal signals (TSS/+150bp) over gene body signals (+250/+2250bp). RNA sequencing (n = 3 mice per condition) and PRO sequencing (n = 3 mice per condition) were conducted with separate mice cohorts. Data are reported in heatmaps as z-score log2 fold changc. **D**, Immunoblotting of Notch signaling co-activator Rbpj-k in muscle stem cells 48h after plating. KMT5a and H4K20mel, and total H3 were used as knockout and loading controls, respectively. **E, F)** Immunoblotting (E) and quantification (F) of MuSCs following Kmt5a catalytic inhibition. Kmt5a inhibitor (Kmt5a i) reduces H4K20mel without affecting Kmt5a levels (n = 3). G) mRNA levels of myogenic genes and Notch target genes following Kmt5a i treatment versus vehicle (veh). Data are reported as normalized fold-changc ± s.d (n = 3). **H-J**, Notch signaling rescue experiments using muscle specific double mutant mice whereby following induction the Notch intracellular domain is overexpressed in the ROSA locus and *Kmt5a* is deleted (Pax7^CrcERT2−^; Kmt5a ^FL/FL^; ROSA^NICD^ [Kmt5a^KO^;NICD]); To confirm the mouse works as intended, we assessed *Kmt5a* and Notch target genes mRNA levels (Figure S6A). Muscle regeneration experiment muscle stem cell counts (Pax7’DAPr) per fiber (Laminin) at homeostasis six weeks after last the tamoxifen injection (H). Number of MuSCs (Pax7+) and quiescent MuSC (Pax7+ KÍ67−) were then quantified. For all violin plots, data represent biological replicates. Statistical significance was determined using Welch’s t-test (F, G) or one-way ANOVA (I. J), and exact p-values and adjusted p-values (q) are reported in the figure.

To identify the mechanisms by which Kmt5a affects MuSC quiescence maintenance, we conducted a suite of transcriptomic assays. Because the Kmt5a^KO^ MuSC number declined stochastically over time (Fig. 2D), we used single-cell RNA sequencing to assess the dynamic transcriptional changes in Kmt5a-null MuSC fate over time. Unbiased clustering and UMAP reduction showed that wild-type cells were significantly different from the Kmt5a^KO^ MuSCs. As expected, the Kmt5a^KO^ MuSC population 2-weeks post deletion displayed a different transcriptomic profile than the 6-weeks post deletion MuSCs, which surprisingly resembled 2-years knockout MuSCs (Fig. 3A). Pseudo-time analysis show that a small subset of MuSCs exist only 2-weeks after *Kmt5a* deletion and present upregulation of myogenic differentiation genes *Myog* and *Mymk*, along with the cell cycle arrest gene *Cdkn1c* (Fig. 3B, and Figure S5C-E). In every mutant sample, we observed a drift in the Kmt5a^KO^ MuSC population, with large changes in quiescence regulatory genes, such as Notch Signaling^28^, Mitophagy^22^, and ECM-receptor interactions^29^ (Figure S5A). Within this population, pseudo-time analysis showed that several key Notch-related genes and downstream targets (Notch, Jag1, Numb, and Rbpj) were downregulated over time, along with other myogenic markers (*Pax7, Sdc4*, and *Spry1*), consistent with the loss of quiescence (Figure S5C and E). Next, we combined Bulk RNA-seq with PRO-seq to compare wild-type and Kmt5a^KO^ MuSCs. RNA-seq was used as a measure of total mRNA, whereas PRO-seq was used to determine dynamic transcriptional changes at base-pair resolution^30^. Most of the significantly altered genes within the Notch signaling pathway were regulated through promoter-proximal pausing (Fig. 3C). The pausing index significantly increased for several key Notch effectors (e.g., *Rbpj* and *Dll1*) and downstream targets (e.g., *HeyL*, *Hes6*, *Dtx4*, and *Snw1*), which correlated with significant gene repression. We also found the opposite to be true, as a decrease in the pausing index at *Dll4* and *Jag2* promoters, both Notch1 ligands that are usually repressed, correlated with increased expression^31^. Overall, *Kmt5a* deletion accounted for > 80% of the altered Notch pathway gene expression, highlighting Kmt5a as a potential master transcriptional regulator of Notch signaling in quiescent MuSCs. This translated into lower levels of Rbp-jκ protein, a critical regulator of MuSC quiescence^32^ (Fig. 3D). Genetic deletion of *Kmt5a* in MuSCs did not immediately result in the repression of Notch genes (Figure S6A) despite the loss of detectable Kmt5a binding at *the Rbpj* TSS (Figure S6B). Instead, Notch target genes repression was observed at the earliest timepoint in our scRNA analysis and onward (Figure S5D and S5E). This was in line with the progressive loss of MuSC quiescence observed in the mutants during homeostasis (Fig. 2D). Therefore, we wondered whether Kmt5a-mediated regulation of Notch genes was catalytically dependent. Since H4K20me1 was previously shown to promote transcription through RNA Polymerase II release during promoter-proximal pausing, we tested whether loss of H4K20me1 might be a necessary step for Notch genes repression^33^. We used a well-characterized selective Kmt5a inhibitor^34^ that resulted in rapid H4K20me1 decline without affecting Kmt5a levels (Fig. 3E, F). Upon catalytic inhibition of Kmt5a in MuSCs, the transcript levels of *Hes1, Hey1, Notch1*, and *Rbpj* were significantly decreased without affecting myogenesis genes (Fig. 3G). We concluded that Kmt5a is an epigenetic regulator of Notch genes transcription.

Based on these results, we hypothesized that restoring Notch target gene expression could rescue Kmt5a-mediated loss of quiescence. To test this hypothesis, we crossed MuSC-specific Kmt5a^KO^ mice with the ROSA^NICD^ mouse line^35^. Following tamoxifen-mediated induction, MuSCs are both null for *Kmt5a* and overexpress the Notch intracellular domain (NICD), a DNA-binding domain that promotes the expression of Notch target genes. NICD overexpression rescued the expression of key Notch target genes such as *Rbpj* and *Hes1* without altering *Kmt5a* expression (Figure S6C). Restoring Notch signaling in Kmt5a^KO^ MuSCs prevented their loss and decreased the number of Ki67 + cells, suggesting that it preserved quiescence (Fig. 3H–J). However, skeletal muscle regeneration was still severely impaired compared to that in wild-type mice, analogous to that observed in Kmt5a^KO^ mice, and we did not detect surviving MuSC at 21dpi (Figure S6D, E). To further assess the fate of MuSCs derived from double-mutant mice, we cultured freshly isolated cells and assessed their survival. Similar to the single mutant Kmt5a^KO^ cells, the double mutant cells displayed a decline in cell survival after 72 h of culture (Figure S6F). These results indicate that, while Kmt5a epigenetically controls MuSC quiescence maintenance through Notch signaling, subsequent cell survival after quiescence exit involves a distinct mechanism.

### Kmt5a safeguards MuSCs from ferroptosis.

We took advantage of our transcriptomic data to further assess the cell fate and determine the fate of the subpopulation of Kmt5a^KO^ MuSCs that exit quiescence. Curated enrichment analysis for significantly modified genes highlighted ferroptosis in both the RNA- and PRO-seq datasets, whereas the pausing index was enriched for Notch signaling and several cancer-related pathways, consistent with the observations in Fig. 3 (Figure S7A-E). Ferroptosis is a unique form of programmed cell death regulated by iron-mediated increases in reactive oxygen species that lead to lipid peroxidation, ultimately resulting in membrane rupture, cell death, and release of pro-inflammatory factors^36^. Mechanistically, this process is not completely understood and is the focus of extensive investigations for its potential contribution to cancer, aging, and frailty^37,38^. Owing to recent advances in this field, ferroptosis is associated with several molecular, morphological, and biochemical hallmarks of ferroptosis^39^. The transcriptional signature in Kmt5a-null MuSCs suggested a pro-ferroptotic fate with a pathway activation z-score of 4.15 and an enrichment score of 44.06 (*Adj. p value* = 0.02650) (Figure S7A). We did not find a strong correlation between ferroptosis markers and the pausing index (Figure S7F), suggesting that ferroptosis might not be regulated through promoter-proximal pausing. Interestingly, most of the genes silenced in Kmt5a^KO^ MuSCs were anti-ferroptotic and either involved in iron processing and export (*Ftl1 and Pcbp2*) or inhibition of lipid peroxidation and cellular damage (*Gclc*, *Gss*, *and Gpx4*). In contrast, the upregulated genes were either markers of ferroptosis (*Ptgs2*, *Cybb*, *and Hmox1*) or genes that promoted intracellular iron import (*Tfrc*) (Figure S7F).

**Figure 4:**
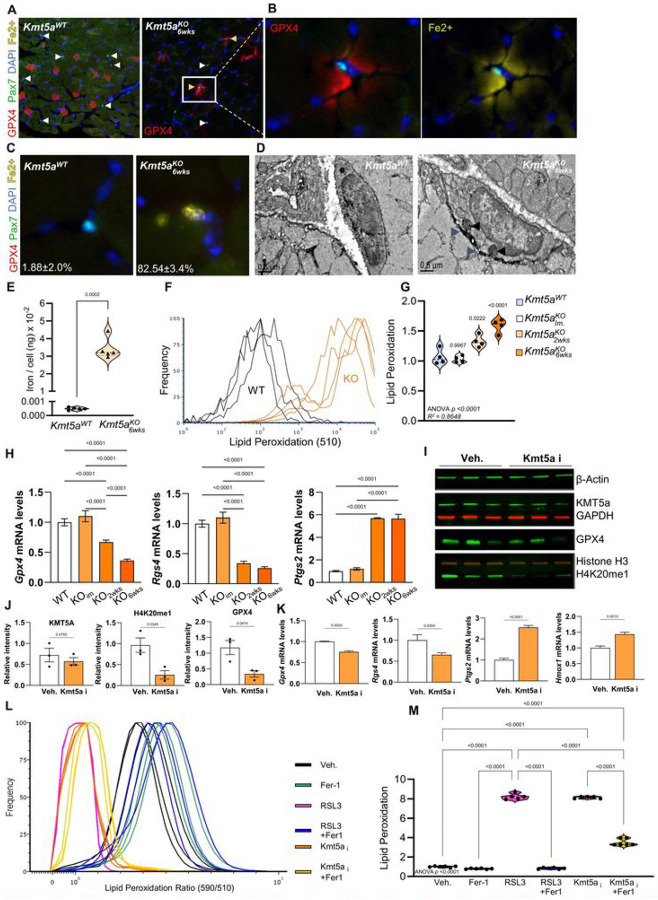
*KmtSa* deletion causes to ferroptosis in muscle stem cells via dysregulation of iron metabolism. **A. B)** Immunofluorescence urates showing GPX4 staining in tibialis anterior muscles of wildtypc (a)and *KmtSa* knockout (b) micc. White arrows indicate muscle stent cells, while the yellow arrow indicates muscle stem cells residing in an iron-rich pockct (~50%). **C)** Quantification of muscle stem cclls with high levels of labile iron (Fc^2+^). Results arc reported as percentages (mean i s.c.m. n 5 micc per condition» **D)** Representative electron micrographs of a wildtypc quiescent muscic stem cell and a Kmt5a*° musck· stem cell showing features of activation and ferroptosis. **E)** ICP-MS quantification of elenvcntal iron in muscle stem cells. Total iron was normalized to cell numbers. Data ponus arc reported as average of replicate (n 5 micc per condition). **F, G)** Quantification of lipid peroxidation in muscle stem cclls. Flow cytometry plot shows a shift in SlOnrn signals in mutant muscic stem cells. Inverted ratiometric signals of 590/510 was calculated to report lipid peroxidation in each cell (<i); data points are reported as average of replicate (n = 4 mice per condition). **H)** qPCR quantification of ferroptosis markers *(ipx4, Rgx4* and *Ptgs2* at various timcpomt post-dclction of *Kmt5a* in MuSCs I. J) ImmunoMot ami quantification for KMTSa, ll4K20mel ami GPX4 relative to controls in response to DV1SO or KmtSa i KmtSa catalyiie inhibitor UNC0379 reduced H4K20mel. Kmt5a was normalized to GAPIMl, ll4K20mel was normalize«! to llistonc II?. and GPX4 was normalized to Aclin. **K)** qPC. R quantification of fcrroptosis markers *Gpx4, Rgs4. Plgf2*. and *llmoxl* in response to KmtSa i. For qPC’R data, gene expression was normalized to the average levels of *B2M*. *TBP* and *PPIA* and arc reported as normalized fold-ehange t s.d.. **L, M** Quantification of lipid peroxidation in response to drug treatments. Histogram shows the intensity of ratio-metric signal <590/510) for the lipid peroxidation probe in live cells (L). Violin plots represent the inverted ratio-metric signal nonnalizcd to vehicle <M). For all violin plots, data represent biological replicatcs. Statistical analyses were performed using Welch’s t-tcst (E. J. K) and one-way ANOVA (G. H. L) and cxact p-values and adjusted p-valucs (q) arc repotted in the figure.

Based on these results, we hypothesized that Kmt5a-deficient MuSCs would exhibit impaired iron metabolism, aberrant accumulation of intracellular iron, and increased levels of lipid peroxidation, resulting in cell death by ferroptosis (Figure S7G). To test this hypothesis more directly, we first characterized MuSC sensitivity to ferroptosis using gold standard compounds to induce ferroptosis (erastin and RSL3), along with Ferrostatin-1 (Fer1), which traps lipid radicals to rescue the effects of erastin and RSL3 (Figure S8)^36^. Both erastin and RSL3 induced a canonical ferroptotic response, with decreased cell viability, increased ROS production, and enhanced lipid peroxidation, which were rescued by Fer1 treatment (Figure S8). MuSCs were more sensitive to RSL3 exposure, perhaps because they directly target GPX4, which plays a critical role in preventing lipid peroxidation, and thus provides a major safeguard against ferroptosis. Interestingly, *Gpx4* was among the most repressed genes identified in our scRNA-seq analysis (Figure S5B), particularly in the population of Kmt5a-deficient MuSCs that exhibited a cell cycle re-entry molecular signature. *In vivo*, we found that GPX4 was expressed in *tibialis anterior* muscle fibers with a small cross-sectional area, implying a potential role for GPX4 in type 2A fibers^40^ (Fig. 4A). Interestingly, Kmt5a^KO^ MuSCs were virtually devoid of GPX4 and exhibited iron-rich pockets in the vicinity of the MuSC niche (Fig. 4B and 4C). Less than 2% of MuSCs from wild-type mice exhibited detectable levels of intracellular iron (Fe^2+^), whereas over 80% of Kmt5a^KO^ MuSCs exhibited accumulation of iron foci (Fig. 4C). Electron microscopy further suggested that Kmt5a^KO^ MuSCs displayed hallmarks of ferroptosis, including cell swelling, plasma membrane blebbing and rupture (blue arrows), increased mitochondrial content, and intracellular iron accumulation (black arrows) (Fig. 4D). ICP-MS was used to measure the total iron content in freshly sorted MuSCs. Kmt5a^KO^ MuSCs had. ~ 3.4×10^− 2^ng of iron per cell, which was nearly 70-fold higher than that measured in wild-type MuSCs (Fig. 4E). Consistent with these findings, the Kmt5a^KO^ MuSCs exhibited higher levels of lipid peroxidation (Fig. 4F), which progressively increased over time (Fig. 4G). Likewise, genetic *Kmt5a* deletion resulted in the progressive rise of a pro-ferroptotic transcriptomic signature in Kmt5a^KO^ MuSCs, including repression of *Gpx4* and *Rgs4* and higher expression of *Ptgs2*^41,42^ (Fig. 4H).

Next, we wondered if Kmt5a catalytic inhibition could promote ferroptosis. Kmt5a inhibition was accompanied by signs of ferroptosis at both the cellular and molecular levels. Dosage under the inhibitor IC_50_ ([C] = 4uM < IC_50_ = 7.3uM), while still significantly decreasing H4K20me1, was seemingly lethal in under 24h (Fig. 4I, 4J, S8J and S8K). Loss of H4K20me1 following treatment was accompanied by decreased GPX4 protein and mRNA levels, as well as repression of *Rgs4* and increased *Ptgs2* and *Hmox1* levels, mirroring the effects observed in RSL3 treated cells (Fig. 4I–K and S8H-L). Kmt5a inhibition also significantly increased lipid peroxidation, which was reduced when co-treated with Fer-1 (Fig. 4L and 4M), further supporting that cell death promoted by Kmt5a inhibition is ferroptosis.

Taken together, these results suggest that Kmt5a activity prevents premature ferroptotic MuSC death by epigenetically regulating key genes involved in iron metabolism and antioxidative activity upon cell cycle re-entry.

### Epigenetic erosion of H4K20 drives MuSC population drift toward ferroptosis during aging.

**Figure 5:**
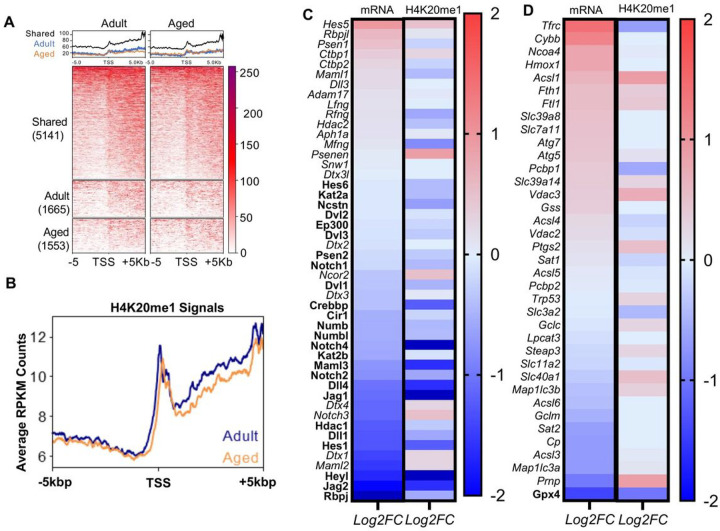
Loss of H4K420mel and Kmt5a in aged Muscle stem cells decrease Notch Signaling and activate Ferroptosis. **A, B)** H4K20mel CUT&Tag in adult and aged muscle stem cells. (A) Heatmap of RPKM normalized sequencing reads centered on TSSs near H4K20mel sites unique to young and aged muscle stem cells or shared between them. The numbers of TSSs in each group are labeled. (B) Metaplot comparison of RPKM averaged in 50bp bins around all TSS. **C, D)** Transcriptional and H4K20mel changes for Notch Signaling genes (C) and Ferroptosis genes (D). Heatmaps are organized by descending gene expression in aged Muscle stem cells are reported as fold-change of z-score for RNA sequencing and H4K20mel CUT&Tag. RNA sequencing and CUT&Tag were performed using different sets of mice and different times (n = 3 mice per condition, per experiment).

Although not previously molecularly linked, increased pro-inflammatory features, loss of MuSC quiescence, and premature death are the hallmarks of skeletal muscle aging. While ferroptosis is an incompletely understood form of programmed cell death, especially in stem cell homeostasis, recent evidence indicates that ferroptosis is implicated in the pathogenesis of various age-dependent disorders^38^. Ferroptosis shares most, if not all, of the hallmarks of aging. Here, we aimed to confirm whether the key findings observed in Kmt5a^KO^ MuSCs are physiologically relevant to aging. Specifically, we assessed the contribution of Kmt5a and H4K20me1 to the age-dependent decline in MuSC number and function. Since we found that aged MuSCs displayed lower levels of both H4K20me1 and Kmt5a than MuSCs from young mice (Fig. 1G–M), we used flow cytometry to assess the activation status of aged MuSCs upon loss of H4K20me1 at the single-cell level. Flow cytometry analyses revealed that while virtually all quiescent MuSCs from young mice exhibited high levels of H4K20me1 (Ki67- H4K20me1^high^), an age-dependent reduction in this population of MuSCs suggests H4K20me1 erosion (Fig. 5a), consistent with the information theory of aging^4^. Furthermore, more than 95% of MuSCs positive for the cell cycle entry marker Ki67 showed lower levels of H4K20me1, consistent with decreased H4K20 methylation being necessary for quiescence exit, and thus a hallmark of MuSC early activation (Pax7^+^ Ki67^+^ H4K20me1^low^) (Figure S9A).

To further understand how Kmt5a epigenetically controls MuSC fate, we mapped H4K20me1 in MuSCs from young and aged mice using CUT&Tag (Figure S9B). Consistent with previous studies using somatic cells, we found that H4K20me1 is a broad epigenetic mark with narrow peaks mostly localized near the TSS of genes and broader peaks spanning over the body of expressed genes (Fig. 5A, B, and Figure S9C). To gain further insights into H4K20me1 regulation, we integrated H4K20me1 maps with our RNA-seq data and found that H4K20me1 was associated with transcriptional status regardless of age (Figure S9D). Term analysis revealed that genes with a loss of H4K20me1 at their TSS were associated with the cell cycle and chromatin organization, while genes that gained H4K20me1 signal were notably enriched for the activation of cell death (Figure S9E). Further, GSEA identified key processes that were also identified in Kmt5a^KO^ MuSCs, including Notch signaling, P53 signaling, and myogenesis (FDR < 0.1) (Figure S9F-H). Most notably, we confirmed that Kmt5a acts as a master epigenetic regulator of Notch Signaling, as over 78% of Notch genes were repressed in aged MuSCs in an H4K20me1-dependent manner with a Pearson correlation *p-value* of 0.0023 (Fig. 5C; bold). Although Notch relies on signaling-based gene regulation, ferroptosis is primarily a metabolic process. Therefore, the role of H4K20me1 in the transcriptional regulation of ferroptotic genes was unclear, except for a few key genes, notably *Gpx4*, which exhibited the most significant reduction in transcript levels (Fig. 5D, bold). Instead, these data highlight that aged MuSCs display signs of a pro-ferroptotic transcriptional signature with repressed Glutathione Metabolism genes such as *Gclm* and *Gclc*, enhanced iron import, and handling with elevated iron-responsible element genes such as *Tfrc, Fth1*, and *Ftl1*, as well as canonical markers of ferroptosis, such as high *Ncoa4, Hmox1*, and *Ptgs2*.

**Figure 6:**
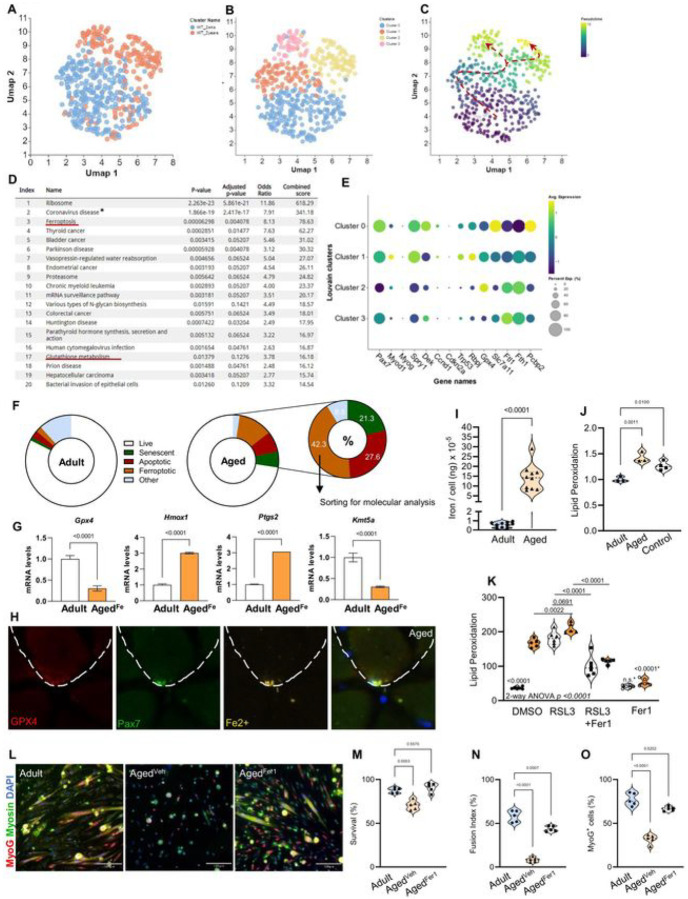
Identification and characterization of a novel ferroptotic subpopulation in aged MuSCs. **A-C)** High-dcpth single cell RNA sequencing analysis strategy: Muscic stem cclls from adult (blue) and aged (orange) were identified and plotted with UMAP (Λ). Unbiased clustering separated adult and aged cells into 4 clusters (2 lor each age) (B) prior to running pscudotimc analysis to assess cell trajectory (C). **D)**, Enrichment analysis of 500 most altered genes as a function of velocity and pscudotime trajectory highlighting Ferroptosis and Glutathione Metabolism (red underlined). ‘Coronavirus was biased by the abundant number of ribosomal genes. **E**, Dot Plot for Louvain metadata clusters of muscle stem cells showing average expression and percent expression per cell for key genes including myogcncsis. muscic stem ccll aging canonical hallmarks. Notch genes, and ferroptosis genes. **F**, Donut plot representing a selection of muscle stem cell fate based on flow cytometry analysis (n = 5 mice per condition). Live cells were negative for all markers, senescent cells were SPiDER^+^, apoptotic cells were Annexin V^+^, ferroptotic cells were Lipid Peroxidation”‘^·, other cells were DAPI but negative for other markers. **G**, Lipid Pcroxidation^h,^*^h^ muscle stem cells (Agetl^K^) were sorted for mRNA analysis to confirm a ferroptotic signature similar to Cluster 3. **H**, Representative picture of an aged muscle stem cell with intracellular labile iron and niche GPX4.1, J, Abondance of total elemental iron per cell (I) and lipid peroxidation (J) in adult and aged muscle stem cells. To avoid additional introduction of ccll stress bias and validate lipid peroxidation changes in aged cclls. we used a glutathione depiction mouse model (Nrf2^KO^;GCLC^KO^) to naturally increase lipid peroxidation, therefore bypassing the need for a compound (i.e.: RSL.3) or iron overload. **K**, Lipid peroxidation in muscle stem cells. Cells were isolated from adult and aged mice (n 5 male mice per condition), plated for 4hours, and harvested 20 hours after treatment. **L–O**, Muscic stem ccll survival (M). myogenic potential (Ö). and fusion competence (N) in response to radical trapping drug Fer-I. For all violin plots, data represent biological replicates. Statistical analyses were performed using Welch’s t-test (G. I and J). one-way (M-O), and two-way ANOVA (K) and exact p-values and adjusted p-values arc reported in the figure.

To further characterize the possible presence of a pro-ferroptosis MuSC population during aging, we performed high-depth scRNA-seq on MuSCs from adult and aged mice (Fig. 6A–C). Enrichment analysis for the most altered genes highlighted *Ribosome* as the top pathway, indicative of the high transcriptional and translational turnover associated with MuSC activation. We identified *Ferroptosis* and *Glutathione Metabolism* as among the pathways most enriched in MuSCs from aged mice (Fig. 6D). Pseudotime analysis identified a novel subpopulation of aged MuSCs (Cluster 3) displaying hallmarks of ferroptosis such as low levels of *Gpx4*, *Slc7a11*, and *Pcbp2*, and high *Ftl1* and *Fth1* (Fig. 6C–E). Both aging clusters (2 and 3) displayed similar aging signatures, such as decreased quiescence gene expression (*Pax7, Spry1*, and *Rbpj*). However, unlike the canonical aging cluster 2^43,44^, the ferroptotic cluster (cluster 3) differentiated itself by displaying no signs of premature commitment (*MyoD and MyoG*) or premature senescence (*Cdkn2a and Trp53*). Interestingly, *Kmt5a* was also significantly downregulated along with *Gpx4*, mostly in cluster 3, suggesting that the loss of *Kmt5a* could directly contribute to enhanced ferrosensitivity (Fig. 5E and Figure S9I, J).

To better understand the extent to which MuSC ferroptosis contributes to skeletal muscle aging, we performed flow cytometry on adult and aged MuSCs, and assessed their viability, cell fate, and different forms of programmed cell death^12,43^. As previously shown, only a small percentage of aged MuSCs entered senescence prematurely (as opposed to geriatric MuSCs)^43^ (Fig. 5F; SPiDER^+^)^11^. The remaining and the majority of dying aged MuSCs were skewed toward ferroptosis (~ 42%; AnnexinV^−^; Lipid Peroxidation^High^), with fewer cells undergoing apoptosis (~ 27%; AnnexinV^+^), indicating that ferroptosis is the dominant mode of regulated cell death in aged MuSCs (Fig. 6F). Upon sorting, ferroptotic aged MuSCs expressed very low levels of *Gpx4* and *Kmt5a*, but high levels of *Hmox1* and *Ptgs2*, consistent with a canonical pro-ferroptotic fate response and similar to cluster 3 (Fig. 6E and G). Upon assessing ferroptosis *in vivo*, we found that < 10% of aged MuSCs displayed faint levels of cytoplasmic GPX4 in concert with an abundance of intracellular iron (Fig. 6H and Figure S9K), which was not observed in MuSCs from young mice. This suggests that while aged MuSCs are defective in processing iron and fail to resist ferroptosis, some aged MuSCs retain the capacity to express GPX4, thus suggesting a reversible process. Similar to Kmt5a^KO^ MuSCs, a significant proportion of aged MuSCs displayed high intracellular iron levels (> 31%), with a concentration far exceeding that measured in MuSCs from young mice (Fig. 6H, I). Interestingly, labile iron accumulation has also been observed in aged hematopoietic stem cells^45^. While circulating iron is largely depleted with age (anemia), labile iron is often enriched in the aging tissues. Iron enrichment in aged skeletal muscles is believed to contribute to both functional and regeneration impairments as well as forms of muscle wasting, such as sarcopenia^37^. Consistent with reports showing that increased intramuscular iron is associated with increased lipid peroxidation^46^, we found that freshly isolated aged MuSCs displayed a 1.5-fold increase in lipid peroxidation during homeostasis (Fig. 6J). To avoid stress response bias, we used MuSCs from Rosa^CreERT2^;Nrf2^f/f^;Gclc^f/f^ mice^47,48^ treated with tamoxifen (50 mg/kg i.p. for five days), which produces Nrf2-Gclc double-KO cells, as a control, since a lack of glutathione in these cells naturally increases ROS production and lipid peroxidation^49^. Notably, *Glutathione Metabolism* was also enriched in aged MuSCs (Fig. 6D), consistent with altered glutathione metabolism, which is a hallmark of stem cell ageing^50^. Upon plating, the rate of lipid peroxidation dramatically increased (> 4.5-fold increase) in aged MuSCs compared to that in younger cells, highlighting the enhanced sensitivity of aged MuSCs to oxidative and replicative stress outside their niche (Fig. 6K). Although RSL3 treatment increased lipid peroxidation to comparable levels in MuSCs from both young and aged mice, Fer1 co-treatment rescued these effects. Importantly, Fer1 treatment alone prevented the increase in basal levels of lipid peroxidation observed in aged MuSCs (Fig. 6K) and significantly enhanced both their viability and myogenic potential (Fig. 6L–O). Taken together, these results demonstrate that ferroptosis is a dominant contributor to the decline in MuSC numbers with age, where activation of the ferroptotic program is triggered by the loss of Kmt5a.

Lastly, because human hematopoietic stem cells were recently shown to be selectively vulnerable to ferroptosis^51^ and labile iron accumulation was observed in aged hematopoietic stem cells^45^, we wondered whether age-associated death by ferroptosis is a shared fate across other adult stem cells. As previously described, we found that both short- and long-term HSCs were enriched in the aged bone marrow (Figure S10 A, B), with long-term HSCs displaying myeloid-bias CD41, a hallmark of HSC aging (Figure S10C). Upon sorting long-term HSCs from young and aged mice, we found that only aged LT-HSCs displayed enhanced lipid peroxidation (Figure S10D).

Taken together, these data indicate that lipid peroxidation and cell death by ferroptosis are important mediators of stem cell aging, and warrant further exploration.

### Prevention of systemic inflammation averts muscle stem cell aging.

**Figure 7:**
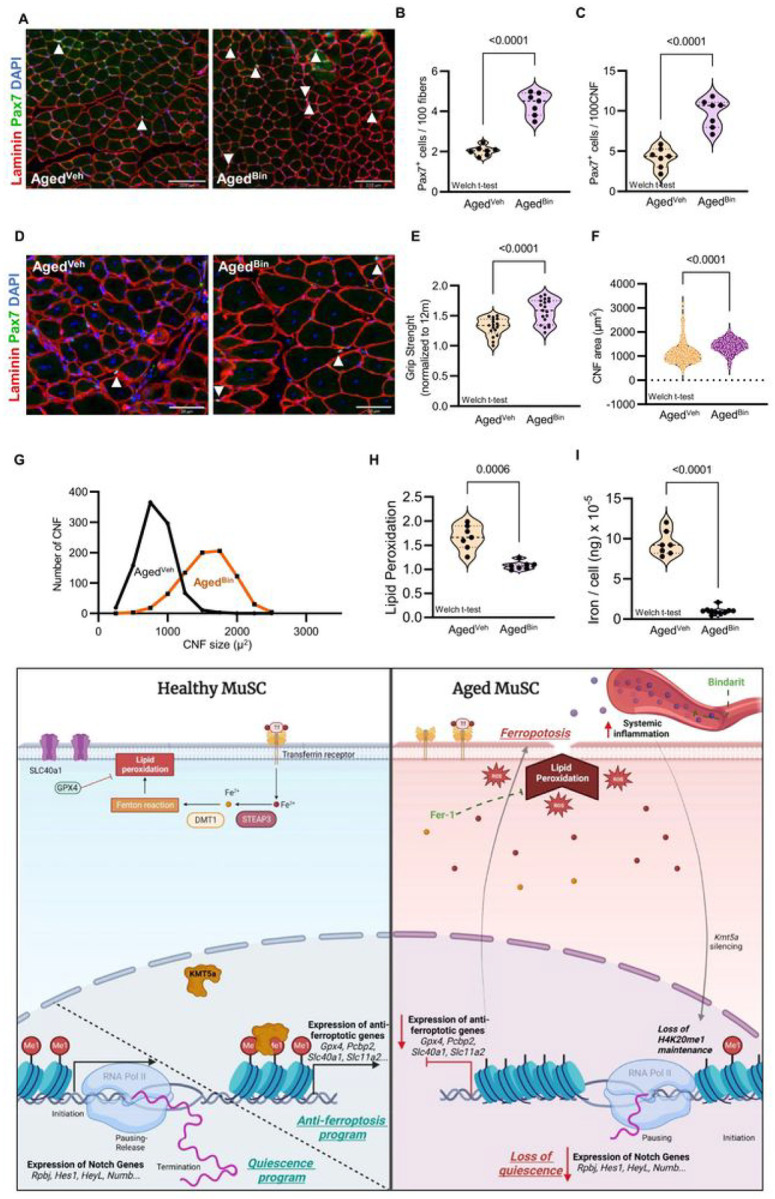
Lifelong inhibition of inflammation rejuvenates skeletal muscle and improve health- and lifespan. **a-d**, Quantification of MuSC numbers at homeostasis (a. b) and 21-day post-regeneration (c. d) in aged mice. Vehicle (Veh) and Bindarit (Bin; 30mg/kg/wk 1P) treated mice were injected weekly between 12 month of age and 24–30month of age. e. Grip strength measurement after regeneration normalized to adult mice, (c) Histological evaluation of muscle regeneration, **f**, **g**. Quantification of the rcgcncra.ed myofiber size (f) and size distribution (g) in the TA muscle, **h**, **i**. Quantification of lipid peroxidation (h) and single cell average iron content (i). **j**. Proposed model for Kmt5a-mcdiatoi disruption of quiescence followed by ferroptosis activaion in aged muscle stem cells. Loss of Kmt5a and downstrean effects are triggered by accumulation of circulatory pro-inflammatory factors and can be reversed by Bindarit. Welch’s t-test. Rach dot represents a mouse replicate. *p values* are reported in the figure.

Finally, because we found that *Kmt5a* levels decreased in MuSCs in response to both distal injury and ectopic induction of acute systemic inflammation, we assessed whether long-term inhibition of these pro-inflammatory molecules could restore *Kmt5a* levels in MuSCs, prevent Kmt5a-mediated loss of quiescence and ferroptotic cell death, and improve skeletal muscle homeostasis and regeneration during aging (Fig. 7). First, we confirmed that aged mice treated with an anti-inflammatory drug specifically targeting our pathway of interest (Bindarit 1/wk IP 30 mg/kg [12to24–30mo]) exhibited significantly diminished cellular and molecular inflammatory profiles similar to those observed in young mice (Figure S11A-K). As expected, Bindarit treatment restored *Kmt5a* levels in MuSCs from aged mice back to young levels (Figure S11L). *In vivo*, the number of MuSCs was significantly improved both at homeostasis and after regeneration compared to vehicle-treated aged mice (Fig. 7A–D), complemented by enhanced muscle repair and restoration of muscle strength after injury (Fig. 7D–G). Finally, both intracellular iron and lipid peroxidation significantly decreased in MuSCs derived from Bindarit-treated aged mice (Fig. 7H and I). Unexpectedly, we observed that Bindarit-treated mice were leaner (Figure S11M) and had an extended lifespan (Figure S11N). Improvement in muscle regeneration was not observed in adult mice, despite the presence of fewer circulatory monocytes (Figure S12A-F). It was also not detrimental, possibly benefiting from circumstances with chronic inflammation only, as previously reported^52–54^.

## Discussion

Our study provides insight into the interplay between inflammation, epigenetics, and stem cell fate in the context of aging. Through the use of genetic models and multi-omics analysis, we propose a novel mechanism whereby the decline of Kmt5a and its associated histone mark H4K20me1 in aged muscle stem/progenitor cells (MuSCs) triggers premature exit from quiescence, ultimately resulting in cell death by ferroptosis.

Genetic deletion of *Kmt5a* causes disruption of quiescence and loss of MuSC numbers over time. The loss of the Kmt5a-mediated histone mark H4K20me1 in MuSCs led to large transcriptional changes, including Notch target gene silencing, which functionally leads to quiescence exit. Upon activation, MuSCs lacking the ability for *de novo* H4K20me1 deposition prematurely die through ferroptosis, a yet uncharacterized form of regulated cell death in MuSCs. Yet, ferroptosis was identified as the dominant form of regulated cell death in aged MuSCs. The roles of ferroptosis and iron metabolism in MuSCs, especially in aging, are currently unclear. Iron metabolism and ferroptosis contribute to muscular pathogenesis, such as sarcopenia and rhabdomyosarcoma, which have recently gained traction but remain largely unknown^44^. MuSC-specific deletion of *Tfrc*, a receptor that mediates the intracellular import of transferrin-bound iron, impairs skeletal muscle regeneration by causing irreversible depletion of the pool and further cell-autonomous defects in proliferation and differentiation^46,55^. Surprisingly, the study showed that preventing Tfrc-mediated import of iron in MuSCs leads to labile iron accumulation and lipid peroxidation in the regenerating muscles, seemingly inducing ferroptosis within the regenerating muscle. Taken together, these observations suggest that not only iron is an important component of myogenesis^56^, but iron scavenging by MuSC-derived myoblasts and newly generated fibers is important for preventing aberrant lipid peroxidation and ROS damage in the niche during skeletal muscle regeneration. Although the precise contribution of MuSC iron homeostasis upon exit from quiescence remains to be explored, the role of iron in DNA replication and the G1 to S-phase transition has been suggested previously^57–59^, consistent with the inability of Kmt5a^KO^ MuSCs to progress through the cell cycle, as we found very little EdU incorporation in Ki67 + Kmt5a^KO^ MuSCs. The regulation of iron metabolism, similar to skeletal muscle regeneration, requires complex interactions among various cell types. Therefore, further investigation is required to determine how changes in iron handling affect skeletal muscle aging and resident cell functions.

MuSCs possess the capability to adjust their metabolome, transcriptome, and proteome, transitioning into the G_alert_ phase upon sensing distal injury, which could only occur through circulatory cues^21,60–62^. Interestingly, a recent study has highlighted that MuSCs maintain these changes for weeks after acute skeletal muscle injury^23^. However, the exact mechanism by which MuSCs sustain this enhanced regenerative ability long after the injury has healed, especially when specific systemic factors have receded from the circulatory system, remains unclear. The concept of epigenetic memory provides a plausible explanation of this phenomenon. In the context of adaptive immunity against inflammation and vaccination, this phenomenon illustrates how inflammatory signals can direct long-term memory immunity via intertwined epigenetic and metabolic shifts^63^. Such epigenetic mechanisms allow cells to retain specific transcriptional patterns for extended periods and even transmit these features to progenies^25^. Importantly, Yang et al. recently demonstrated that the loss of epigenetic information is not merely a symptom but also a unified causative agent of aging across mammals^4^, closely intertwined with rising systemic inflammation^64^. However, the aged epigenetic landscape of quiescent stem cells, including MuSCs, remains largely uncharted^7,65,66^, especially regarding the influence of age-related systemic inflammation on the aged MuSC epigenome and transcriptome. Here, we found that several pro-inflammatory cytokines are increased in the aged mouse plasma and are associated with CCR2 signaling activation in aged skeletal muscles. To delineate the role of these cytokines in MuSC aging, we administered a cocktail of chemokines to adult mice and assessed MuSC function. Systemic exposure to CCR2-ligands is sufficient to repress *Kmt5a* in adult MuSCs, causing long-term loss of H4K20me1. In Kmt5a^KO^ MuSCs, or in wild-type cells exposed to systemic CCR2-ligands, the decrease in H4K20me1 was accompanied by G_alert_ features such as swifter cell cycle re-entry, accelerated myogenesis, and enhanced regeneration. Taken together, these cellular and molecular shifts indicated a prolonged injury response shaped by transient inflammatory signals. However, chronic exposure to CCR2-ligands was sufficient to decrease *Kmt5a* and H4K20me1 levels and trigger premature cell death via ferroptosis in MuSCs, partially recapitulating the effects of *Kmt5a* deletion. During regeneration, an acute immune response followed by its resolution is critical for the spatiotemporal progression of myogenesis and successful regeneration^67^. In contrast, chronic inflammation is often implicated in aging and other related disorders. Considering the physiological role of Kmt5a-mediated epigenetic remodelling in response to acute inflammation, we postulated that it serves as an epigenetic switch to prime MuSCs into Galert by silencing Notch target genes. However, sustained inflammation, as observed in aging, could permanently repress *Kmt5a*. This could irreversibly prevent H4K20me1 *de novo* deposition, leading to the detrimental effects observed in Kmt5a-null MuSCs. Thus, we propose that systemic inflammation drives MuSC aging through epigenetic erosion. While these observations are compelling, comprehensive studies are vital to determine whether this phenomenon extends to other aging stem cells^68^.

Kmt5a-mediated deposition of H4K20me1 is critical for mouse development, and deletion of *Kmt5a* results in embryonic lethality before the eight-cell stage^69^. Interestingly, the specific deletion of *Kmt5a* in MuSCs did not result in immediate depletion of the pool. Instead, it mirrored an accelerated stochastic model of aging, whereby MuSC decline is a progressive albeit exponential phenomenon. We hypothesized that the stochastic nature of MuSC decline might correspond to the loss of H4K20me1 over time following *Kmt5a* deletion. Both Kmt5a and H4K20me1 are highly regulated in a cell cycle-dependent manner, enabling proper DNA replication during S phase and safeguarding genomic integrity during mitosis^70^. In somatic cells, *Kmt5a* deletion result in genomic instability and rapid cell death. Since we detected a significant proportion of Ki67 + Kmt5a^KO^ MuSCs, and that most of the Kmt5a^KO^ MuSCs remaining cells were H4K20me1 + six-weeks after deletion, it is possible that mutant MuSCs can survive out of quiescence for as long as they have H4K20me1, supporting the assumption that Kmt5a regulates quiescence and survival via two distinct mechanisms. Further supporting this idea, reactivating Notch signaling with the NICD model prevented the depletion of the Kmt5a^KO^ MuSCs pool at homeostasis, but not following injury. Although restoring Notch could not prevent ferroptosis in Kmt5a^KO^ MuSCs, it is also possible that the overactivation of NICD leads to Kmt5a-independent myogenic impairment^35^. While our data suggest that the loss of H4K20me1, rather than Kmt5a, results in MuSC exit from quiescence and subsequent ferroptosis, further investigation is required to determine what mediates the loss of H4K20me1 following *Kmt5a* deletion. In addition, because Kmt5a-mediated maintenance of monomethylation is required for the subsequent deposition of di- and trimethylation of H4K20, dissociation of the exact contribution of Kmt5a and H4K20me1 requires further investigation. The scarcity of adult stem cells combined with the fleeting status of quiescence makes the study of H4K20 methylation dynamics a challenge that will require both collaborative efforts and the use of new technologies.

Our findings propose a novel mechanistic axis of stem cell aging that links inflammation to Kmt5a-dependent epigenetic regulation of stem cell metabolism, which extends recent findings. Benjamin et al, recently showed that a subset of aged MuSCs is dysfunctional due to high NF-κB-mediated inhibition of NRF2, thus leading to unbalanced glutathione metabolism, which is critical for limiting lipid peroxidation and ferroptosis^49,50^. Given that Bindarit preferentially targets the NF-κB pathways^71^, the observed beneficial effects in Bindarit-treated aged mice are in line with improved glutathione metabolism. Additionally, increased inflammation and iron accumulation are closely associated with human aging. Age-related chronic inflammation leads to increased iron accumulation in various tissues, whereas excess iron promotes inflammation through the generation of reactive oxygen species and activation of pro-inflammatory signaling pathways^38^. This reciprocal relationship between inflammation and iron levels is thought to contribute to the development and progression of age-related diseases. Our data suggest that inflammation may induce epigenetic changes that lead to iron accumulation and potential ferroptotic death in stem cells, which could have important implications for human aging. Because chronic inflammation is a hallmark of aging, it is plausible that the accumulation of iron in stem cells due to inflammation-induced epigenetic reprogramming contributes to age-associated low-grade inflammation. The release of pro-inflammatory molecules associated with ferroptotic death can perpetuate the inflammatory environment, creating a vicious cycle of inflammation, iron accumulation, and ferroptotic death across several aged tissues.

In this study, we present data indicating that age-associated systemic inflammation reprograms MuSCs through epigenetic erosion, leading to the premature loss of quiescence and subsequent cell death by ferroptosis. Our findings directly link aging and inflammation to the loss of Kmt5a function that drives an epigenetic switch to disrupt MuSC quiescence and promote ferroptosis. Our findings provide evidence to suggest that the inflammation-epigenetic-ferroptosis axis has significant therapeutic implications in stem cell rejuvenation. Although iron metabolism is known to contribute to stem cell aging^45^, our study sheds light on this mechanism and highlights its importance in developing new therapeutic avenues. Encouragingly, iron chelators, which are already used for other diseases, can be rapidly translated into stem cell therapy in elderly patients. In addition to emerging evidence on the involvement of ferroptosis in aging disorders, particularly neurodegenerative diseases^72,73^, we present a compelling case for further exploration of the complex interplay between epigenetic erosion-mediated diversity, iron metabolism, and ferroptosis in diverse adult stem cell compartments during aging. Our findings underscore the urgent need for regenerative medicine to further explore the fundamental mechanisms driving these processes with the aim of developing novel therapeutic interventions to combat age-related degenerative diseases. Unraveling these mysteries may pave the way for a new era of personalized regenerative medicine that significantly improves the quality of life of aging individuals and potentially extends human lifespan.

### Limitations of study

Although our study sheds light on the intricate mechanisms linking inflammation, epigenetics, and ferroptosis in MuSC aging, there are several limitations that warrant consideration. First, the aging experiments were restricted to male mice. We also focused on specific genetic strains and a particular age bracket, potentially overlooking the nuances present in different genetic backgrounds and across a broader age spectrum. Further experiments are required to determine whether these mechanisms are conserved in humans. Additionally, our study predominantly emphasized the role of Kmt5a and H4K20me1, and the involvement of other epigenetic marks cannot be ruled out, especially regarding the H4K20me1 loss effects on H4K20me2 and me3. While we believe that the loss of H4K20me1 in MuSCs contributes to quiescence exit, the initiating mechanisms that trigger H4K20me1 erosion during quiescence remain unclear. Furthermore, the complex interactions between various cell types in the regulation of iron metabolism and skeletal muscle regeneration necessitate a more exhaustive exploration. Lastly, while we observed systemic beneficial effects of Bindarit on aged mice, the exact underlying mechanisms of this rejuvenation remain to be fully explored.
